# Low Dehydroepiandrosterone (DHEA) Level Is Associated with Poor Immunologic Response among People Living with HIV/AIDS

**DOI:** 10.3390/jcm11206077

**Published:** 2022-10-14

**Authors:** Eun Hwa Lee, Ki Hyun Lee, Se Ju Lee, Jinnam Kim, Jung Ho Kim, Jin Young Ahn, Nam Su Ku, Jun Yong Choi, Joon-Sup Yeom, Su Jin Jeong

**Affiliations:** Department of Internal Medicine and AIDS Research Institute, Severance Hospital, Yonsei University College of Medicine, Seoul 03722, Korea

**Keywords:** HIV/AIDS, people living with HIV/AIDS (PLWHA), dehydroepiandrosterone (DHEA), immune–virologic discordance

## Abstract

Dehydroepiandrosterone (DHEA) is an adrenal steroid converted to potent androgens. This study aimed to discover the association between serum DHEA levels and immunologic response in people with HIV/AIDS (PLWHA). We enrolled patients aged ≥ 18 years who were treated with combination antiretroviral therapy (cART). We measured CD4+ and CD8+ T-cell counts, HIV-RNA titres, and serum DHEA levels. We assigned each patient to a good- or poor-responder group depending on their CD4+ T-cell counts at study enrolment. Participants with CD4+ T-cell counts > 200/µL were assigned to the good-responder group, whilst those with CD4+ T-cell counts < 200/µL were assigned to the poor-responder group. The participants were followed up for 2 years. The poor-responder group showed lower CD4+ T-cell counts and higher HIV PCR titres at their initial HIV diagnosis and in their 2-year follow-up data. Serum DHEA level was lower in the poor-responder group. Multivariable logistic analysis showed that BMI, initial CD4+ T-cell counts, and serum DHEA level were clinical factors associated with poor immunologic responsiveness to cART in PLWHA. Therefore, DHEA may be used as an indicator of the immunological recovery of PLWHA.

## 1. Introduction

Advances in combination antiretroviral therapy (cART) have enhanced the therapeutic success and prognosis of people with HIV/AIDS (PLWHA). The morbidity and mortality of PLWHA are significantly reduced, making their life expectancy comparable to that of the average population [1]. However, even with appropriate treatment with cART, some PLWHA fail to recover their CD4+ T-cell counts. Despite successful virologic suppression, approximately 15–18% of patients fail to achieve immune recovery, and such patients are referred to as “immune non-responders” or patients with “immune–virologic discordance” [1,2].

The important predisposing factors to poor response to cART are baseline immunosuppression levels and old age [3]. An initial CD4+ T-cell count < 200 is associated with blunted CD4+ T-cell recovery [4,5]. Previous studies have attempted to find supplements, such as zinc or vitamin D, to enhance the immunologic response and prognosis of PLWHA [6,7].

Dehydroepiandrosterone (DHEA) is a naturally occurring steroid mainly produced by the adrenal glands and is converted into potent androgens [8]. Previous studies have indicated that DHEA may have immunomodulatory properties by regulating the cytokine and complement cascades of inflammatory pathways [9]. Preliminary results showed that DHEA might act as an inhibitor of HIV replication [10]. This study hypothesises that serum DHEA is reduced in the poor-immune-responder group compared to PLWHA who successfully recovered CD4+ T-cell counts with cART. We performed a comparative analysis by dividing the good and poor responders and analysed the serum DHEA levels and other clinical factors associated with immune responsiveness. 

## 2. Materials and Methods

### 2.1. Study Population 

All participants were Koreans with HIV infection and were treated with cART at Severance Hospital (Seoul, Korea), a 2000-bed tertiary teaching hospital with an HIV research institution. We prospectively enrolled HIV-infected patients aged ≥ 18 years who were currently receiving cART. Written informed consent was obtained from all the participants. PLWHA without written consent were excluded from the study. From October 2016 to November 2019, serum blood samples were collected from each participant during their regular HIV clinic visits. This study was approved by the institutional review board of Severance Hospital (4-2016-0596).

### 2.2. Study Design and Definitions 

CD4+ and CD8+ T-cell counts and HIV-RNA titres were collected at enrolment and on follow-up visits two years after enrolment in the study. Serum DHEA levels were measured once, at the study enrolment. The data of the initial HIV profile, including CD4+ and CD8+ T-cell counts and HIV-RNA titre at first diagnosis of HIV, were collected by reviewing electronic medical records. According to their CD4+ T-cell counts at the enrolment, the participants were assigned to good- and poor-responder groups. Since PLWHA with CD4+ T-cell counts below 200 have a higher chance of acquiring opportunistic infections, we assigned patients to the good-responder group who showed CD4+ T-cell counts above 200/mm^3^ whilst assigning participants with CD4+ T-cell counts below 200/mm^3^ in the poor-responder group [11]. There is no consensus on an acceptable CD4+ T-cell count recovery range for those on cART [12]. The good- and poor-responder groups were compared using various clinical factors, such as baseline characteristics of patients, underlying medical conditions, and HIV-related profiles such as CD4+ and CD8+ T-cell counts and HIV-RNA viral titres.

### 2.3. Measurement of Plasma DHEA

Each 0.5 cc blood sample was collected in an EDTA tube, centrifuged, and stored at −70 °C until testing. Serum DHEA levels were measured using an enzyme-linked immunosorbent assay (ELISA) kit (LDN GmbH & Co., KG, Nordhorn, Germany). Readings were measured using an ELISA plate reader SpectraMax190 (Molecular Devices, Sunnyvale, CA, USA) at Seoul Clinical Laboratories, Yongin, Korea.

### 2.4. Data Collection

Information on age, sex, weight, waist circumference, body mass index (BMI), underlying medical conditions, type of cART administered at study enrolment, CD4+ and CD8+ T-cell counts, HIV-RNA titres, date of HIV diagnosis, and date of cART initiation were obtained from electronic medical records and laboratory results.

### 2.5. Statistical Analysis

Statistical analysis was performed using Statistical Package for Social Sciences ver. 26 (SPSS, Chicago, IL, USA). Statistical significance was set at *p* < 0.05. All values are expressed as the mean ± standard deviation or median with 25% and 75% values, respectively. Univariate and multivariate logistic regression analyses were performed to evaluate the clinical factors associated with responsiveness to HIV treatment. The receiver operating characteristic (ROC) curve was used to assess DHEA as a predictive marker for a poor immunological response. We used the Youden index to analyse the specific cut-off point of the serum DHEA level to predict poor clinical outcomes. The Biostatics Collaboration Unit of Yonsei University College of Medicine reviewed and certified statistical analysis and data of the study.

## 3. Results

**Figure 1:**
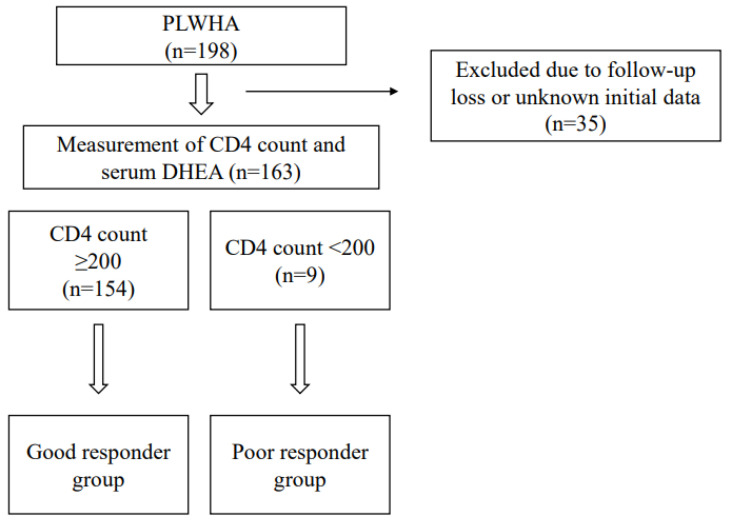
Study flow. PLWHA: people living with HIV/AIDS.

A total of 198 participants were enrolled in this study. Thirty-five patients were excluded from the analysis due to follow-up loss or due to their missing initial HIV profile at first diagnosis (Figure 1). 

Table 1 shows the baseline characteristics of the participants. The average age of the participants was 44.1 years, and 155 (95.1%) were men. The number of participants assigned to the good- and poor-responder groups was 154 and 9, respectively.

Table 2 compares the differences between the two groups. The poor-responder group had a lower BMI, weight, and waist circumference than the good-responder group. Additionally, the poor-responder group showed lower initial CD4+ T-cell count (282.7 vs. 92.4/µL, *p* = 0.001) and CD8+ T-cell count (966.7 vs. 497.3/µL, *p* = 0.025) and higher initial log_10_ HIV-RNA titre (4.7 vs. 5.6 copies/mm^3^, *p* = 0.001) at the first HIV diagnosis. At the two-year follow-up after study enrolment, the HIV profile remained similar to the initial HIV profile at diagnosis. The poor-responder group had a lower CD4+ T-cell count (726.6 vs. 187.4/µL, *p* < 0.0001) and CD8+ T-cell count (821.4 vs. 569.0/µL, *p* = 0.034) than the good-responder group. None of the poor responders had underlying chronic medical conditions such as diabetes mellitus, hypertension, chronic kidney disease, dyslipidaemia, or fatty liver. Whilst the good-responder group showed a fully suppressed HIV-RNA titre, the poor-responder group showed an elevated HIV-RNA titre compared to the good-responder group. Serum DHEA levels were lower in the poor-responder group than in the good-responder group (4.3 vs. 2.9 ng/mL, *p* = 0.013). Both groups showed no significant differences in the duration or treatment of HIV infection. The poor-responder group had a shorter time from diagnosis to treatment (488.6 vs. 83.2 days, *p* = 0.006).

Table 3 compares antiretroviral regimens of the good and poor responders. The poor-responder group tended to take the cART regimen with a greater number of different classes (3.1 vs. 3.0, *p* < 0.001), while a higher percentage of poor-responder participants added protease inhibitors in the regimen than the good-responder group (21.4% vs. 55.6%, *p* = 0.033). 

The clinical characteristics of the poor responders are listed in Table 4 to provide individual information for the poor responders. All of the patients received a cART regimen including integrase inhibitor with or without protease inhibitor. 

Univariable and multivariable logistic regression analyses were performed to analyse the clinical factors associated with poor immunologic response to cART. The univariable logistic regression results in Table 5 demonstrate that factors such as BMI, weight, and waist circumference, which indicate the severity of HIV-related cachexia, are associated with poor immunologic response. The initial CD4+ T-cell count, initial CD8+ T-cell count, and serum DHEA level were also associated with poor immunological responses. In the multivariable analysis, BMI, initial CD4+ T-cell counts, and serum DHEA remained significant clinical factors associated with poor immunological responsiveness.

**Figure 2:**
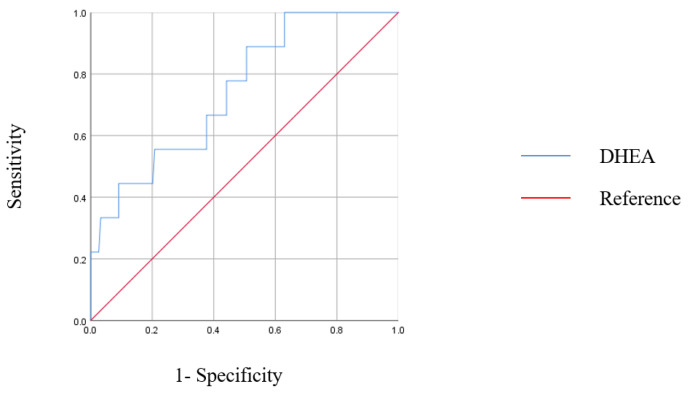
Receiver operating characteristic (ROC) curve for DHEA level in predicting poor immunologic responsiveness. The area under the ROC curve for DHEA was 0.079 (95% CI 0.593–0.901, *p* = 0.013).

To assess the usefulness of serum DHEA levels in predicting an immunologic recovery in PLWHA receiving cART, the ROC curve is displayed in Figure 2. The area under the ROC curve was 0.079 (95% CI = 0.593–0.901, *p* = 0.013). According to the Youden index of the ROC curve, a serum DHEA level of 4.19 ng/mL is the cut-off value for predicting a poor immune recovery in response to cART.

## 4. Discussion

A low initial CD4+ T-cell count and high viraemia are well-known factors related to the poor prognosis of HIV treatment [13,14]. Our study results also confirm that the poor responders had a low baseline CD4+ T-cell count and higher HIV-RNA viraemia state compared to the good responders at HIV diagnosis. Poor responders also presented lower body-mass index, weight, and waist circumference, which supports the previous notion that cachexia is also an important poor prognostic factor in PLWHA [15,16]. Interestingly, the poor responders did not have any chronic medical conditions, but this may be due to the small sample size of a relatively young group (average age 44.9).

Treatment with cART protects numerous PLWHA from progressing to terminal AIDS and reduces the risk of opportunistic infections by improving immune function [13]. However, some PLWHA experienced treatment failure, showing no CD4+ T-cell count recovery even with appropriate treatment with cART and achievement of full viral suppression, also referred to as “immune–virologic discordance” or in many other similar terms by previous research journals [17]. CD4+ T-cell count recovery is essential for the immune function of PLWHA, which is related to the risk of acquiring opportunistic infections and mortality. The previously known risk factors for immune–virologic discordance are low CD4+ T-cell count and high HIV-RNA at diagnosis of HIV, which are indicators of poor treatment response to cART. Among the nine participants who displayed CD4+ T-cell counts below 200/µL, only three participants were able to recover higher CD4+ T-cell counts at the two-year follow-up. 

Many adjunctive therapies have been proposed to improve the immune–virologic discordance of PLWHA, and oral DHEA replacement is one of the suggested therapies [10]. We evaluated the significance of serum DHEA level in PLWHA with poor immune recovery to assess the possibility of the replacement of DHEA as a supplemental method to enhance CD4+ T-cell count as an adjunctive to cART. 

DHEA was first isolated from humans in 1954 [18]. The adrenal cortex is the main organ responsible for DHEA production, but the testes and ovaries also produce DHEA [18,19,20]. Serum DHEA levels are decreased in older patients and those with advanced stages of HIV infection [21,22,23]. Randomised controlled trials have been conducted to improve sexual function, well-being sensation, bone mass density, and cognitive performance by replacing DHEA orally [24]. In a placebo–controlled trial by Rabkin et al., the DHEA replacement was proven useful in treating non-major but persistent depression in PLWHA [25]. Piketty et al. performed a double-blind placebo–controlled trial of oral DHEA (50 mg/day for four months) in patients with advanced HIV stage between June 1995 and November 1996 [26]. The study results confirmed the improvement in physiological and psychological well-being but were unable to show improvements in CD4+ T-cell counts. Additionally, there is an expert opinion that using a higher dose of DHEA (300–600 mg twice daily) may be necessary to improve CD4+ T-cell counts [27].

Currently, there are no guidelines for replacing DHEA with PLWHA. According to our results, oral replacement of DHEA may be considered for patients who satisfy both of the following criteria: (1) serum DHEA level < 4.19 ng/mL and (2) no recovery of CD4+ T-cell count > 200/mm^3^ after treatment with appropriate cART without drug resistance or compliance issues. Since the normal range of DHEA varies according to age and sex, subgroup analysis should be performed in the future to verify the appropriate cut-off serum DHEA values for different age and sex groups. In our study, groups of people displayed low serum DHEA levels but showed good immunologic recovery of CD4+ T-cell counts. Therefore, this finding suggests that low serum DHEA level is not an absolute indication of DHEA replacement. 

Furthermore, serum DHEA level can predict poor immune recovery in PLWHA patients treated with cART. Our journal is the first to use serum DHEA level as a tool to assess therapeutic outcomes. According to the ROC curve, serum DHEA level can be used to predict unfavourable responses to immune recovery. As optimal serum DHEA level varies by age and sex, such clinical factors should be considered when assessing PLWHA for DHEA replacement. 

The limitation of this study is in the small sample size of the poor responders and the absence of clinical results for oral replacement of DHEA. A prospective, case–controlled study comparing the effects of DHEA replacement will help assess the clinical usefulness of DHEA replacement in the poor-responder group. 

Thus far, the previous studies did not report any major side effects due to replacement of DHEA for various purposes [28]. Therefore, therapeutic replacement of DHEA can be incorporated into augmentation of immunologic function in PLWHA. 

## 5. Conclusions

The serum DHEA level can be used to predict poor immunologic recovery in PLWHA treated with cART. Replacement of DHEA in poor responders might be a valuable treatment option for enhancing the immunologic function of PLWHA.

## Figures and Tables

**Table 1:** Baseline Characteristics of the Study Population.

	All Patients
Baseline characteristics	
Number of Patients	163
Age, years	44.1 ± 12.1
Gender, male	155 (95.1)
BMI (kg/m^2^)	23.1 ± 4.1
Weight (kg)	68.5 ± 12.7
Waist (inch)	31.9 ± 5.9
Underlying conditions	
DM	16 (9.8)
HTN	31 (19.0)
Chronic kidney disease	3 (1.8)
Dyslipidemia	28 (17.2)
Fatty liver	9 (5.5)
HIV status	
Known duration of HIV (days)	3168.7 ± 2223.1
Treatment duration with cART (days)	2774.2 ± 2036.9
Time from diagnosis to treatment initiation (days)	28.0 (11.3, 308.0)
Initial CD4+ T-cell count (/µL)	278.0 ± 191.7
Initial CD8+ T-cell count (/µL)	953.4 ± 930.6
Initial log_10_HIV-RNA titre (copies/mm^3^)	4.8 (4.3, 5.4)
Follow-up CD4+ T-cell count (/µL)	694.9 ± 325.5
Follow-up CD8+ T-cell count (/µL)	780.7 ± 399.5
Follow-up log_10_HIV-RNA titre (copies/mm^3^)	0 (0, 0)
Dehydroepiandrosterone (ng/mL)	4.8 ± 3.4

Data are expressed as number (percent), average ± standard deviation, or median (25%, 75%) cART; combinational antiretroviral therapy, BMI: body-mass index; DM: diabetes mellitus, HIV: human immunodeficiency virus; HTN: hypertension; RNA: ribonucleic acid.

**Table 2:** Comparison of the good and poor responders.

	All Patients	Good Responders	Poor Responders	*p*-Value
Baseline characteristics				
Number of Patients	163	154	9	
Age, years	44.1 ± 12.1	44.0 ± 12.3	44.9 ± 8.7	0.711
Gender, male	155 (95.1)	147 (95.5)	7 (87.5)	0.372
BMI (kg/m^2^)	23.1 ± 4.1	23.7 ± 3.6	20.6 ± 2.7	**0.013**
Weight (kg)	68.5 ± 12.7	70.3 ± 12.6	59.6 ± 10.7	**0.013**
Waist (inch)	31.9 ± 5.9	32.1 ± 6.0	29.1 ± 2.4	**0.007**
Underlying conditions				
DM	16 (9.8)	16 (11.0)	0 (0)	0.599
HTN	31 (19.0)	31 (21.2)	0 (0)	0.206
Chronic kidney disease	3 (1.8)	3 (2.1)	0 (0)	>0.999
Dyslipidemia	28 (17.2)	28 (19.2)	0 (0)	0.365
Fatty liver	9 (5.5)	9 (6.2)	0 (0)	>0.999
HIV status				
Known duration of HIV (days)	3168.7 ± 2223.1	3352.1 ± 2222.3	3276.6 ± 2710.9	0.931
Treatment duration with cART (days)	2774.2 ± 2036.9	2898.3 ± 2001.9	3139.3 ± 2587.8	0.808
Time from diagnosis to treatment initiation (days)	28.0 (11.3, 308.0)	488.6 ± 1030.3	83.2 ± 225.5	**0.006**
Initial CD4+ T-cell count (/µL)	278.0 ± 191.7	282.7 ± 187.9	92.4 ± 97.1	**0.001**
Initial CD8+ T-cell count (/µL)	953.4 ± 930.6	966.7 ± 863.0	497.3 ± 325.0	**0.025**
Initial log_10_HIV-RNA titre (copies/mm^3^)	4.8 (4.3, 5.4)	4.7 (4.2, 5.3)	5.6 (5.1, 6.1)	**0.001**
Follow-up CD4+ T-cell count (/µL) *	694.9 ± 325.5	726.6 ± 303.3	187.4 ± 76.9	**<0.0001**
Follow-up CD8+ T-cell count (/µL) *	780.7 ± 399.5	821.4 ± 396.5	569.0 ± 368.1	**0.034**
Follow-up log_10_HIV-RNA titre (copies/mm^3^) *	0 (0, 0)	0 (0, 0)	1.7 (0, 4.1)	**<0.0001**
Dehydroepiandrosterone (ng/mL)	4.8 ± 3.4	4.3 ± 3.4	2.9 ± 1.1	**0.013**

Data are expressed as number (percent), average ± standard deviation, or median (25%, 75%). *p*-values with statistical significance are shown in bold text. * Data at 2-year follow up after study enrolment. cART: combinational antiretroviral therapy; BMI: body-mass index; DM: diabetes mellitus; HIV: human immunodeficiency virus; HTN: hypertension; RNA: ribonucleic acid.

**Table 3:** Comparison of Antiretroviral Therapy in the Good and Poor Responders.

	All Patients	Good Responders	Poor Responders	*p*-Value
Antiretroviral therapy	163	154	9	
Total number of antiretroviral agents	3.1 ± 0.35	3.0 ± 0.02	3.3 ± 0.16	**<0.001**
Integrase inhibitor	125 (81.2)	117 (80.7)	8 (88.9)	>0.999
Nucleoside/nucleotide reverse transcriptase inhibitor	153 (99.4)	144 (99.3)	9 (100.0)	>0.999
Non-nucleoside/nucleotide reverse transcriptase inhibitor	3 (1.9)	3 (2.1)	0 (0)	>0.999
Protease inhibitor	36 (23.4)	31 (21.4)	5 (55.6)	**0.033**

*p* values with statistical significance are shown in bold text.

**Table 4:** Characteristics of the Poor Responders.

	Age (at HIV Diagnosis)	Sex	CD4+ Count (Baseline, /mm^3^)	CD4+ Count (at the End of the Study, /mm^3^)	Antiretroviral Regimen	Duration of HIV Infection(Days)	Duration of HIV Treatment (Days)
Poor responder 1	40	Male	9	290	INI + PI	391	386
Poor responder 2	54	Female	4	137	INI-based	184	183
Poor responder 3	42	Male	116	76	INI + PI	4655	4630
Poor responder 4	29	Male	14	519	INI-based	1	1
Poor responder 5	42	Male	56	201	INI + PI	2854	2844
Poor responder 6	54	Male	229	282	INI-based	7322	6638
Poor responder 7	54	Male	246	133	INI + PI	4712	4690
Poor responder 8	46	Male	8	122	INI based	6372	6372
Poor responder 9	56	Male	150	185	INI + PI	2999	2997

Integrase inhibitor, INI; protease inhibitor, PI.

**Table 5:** Factors associated with CD4+ T-cell count in PLWHA on cART.

	Univariable		Multivariable	
	OR (95% CI)	*p*-Value	OR (95% CI)	*p*-Value
Baseline characteristics				
Age, years	1.006 (0.952–1.063)	0.836		
Gender, male	2.625 (0.287–23.996)	0.393		
BMI (kg/m^2^)	0.863 (0.746–0.999)	**0.048**	0.763 (0.603–0.966)	**0.025**
Weight (kg)	0.908 (0.837–0.984)	**0.019**		
Waist (inch)	0.619 (0.419–0.914)	**0.016**		
HIV status				
Known duration of HIV (days)	1.000 (1.000–1.000)	0.881		
Treatment duration with cART (days)	1.000 (1.000–1.000)	0.521		
Time from Diagnosis to treatment initiation (days)	0.999 (0.995–1.002)	0.342		
Initial CD4+ T-cell count (/µL)	0.990 (0.983–0.997)	**0.005**	0.992 (0.984–0.999)	**0.023**
Initial CD8+ T-cell count (/µL)	0.998 (0.995–1.000)	**0.025**		
Initial HIV-RNA titre (copies/mm^3^)	1.000 (1.000–1.000)	0.055		
Dehydroepiandrosterone (ng/mL)	0.469 (0.230–0.959)	**0.038**	0.311 (0.102–0.948)	**0.040**

*p*-values with statistical significance are shown in bold text. cART: combinational antiretroviral therapy; BMI: body-mass index; HIV: human immunodeficiency virus; RNA: ribonucleic acid.

## Data Availability

All data are available on request.
